# Characterization of the Vaginal Micro- and Mycobiome in Asymptomatic Reproductive-Age Estonian Women

**DOI:** 10.1371/journal.pone.0054379

**Published:** 2013-01-23

**Authors:** Tiina Drell, Triin Lillsaar, Lea Tummeleht, Jaak Simm, Anu Aaspõllu, Edda Väin, Ivo Saarma, Andres Salumets, Gilbert G. G. Donders, Madis Metsis

**Affiliations:** 1 Centre for Biology of Integrated Systems, Tallinn University of Technology, Tallinn, Estonia; 2 Competence Centre on Reproductive Medicine and Biology, Tartu, Estonia; 3 Fertilitas Hospital, Haabneeme, Estonia; 4 Department of Obstetrics and Gynaecology, University of Tartu, Tartu, Estonia; 5 Institute of Biomedicine, University of Tartu, Tartu, Estonia; 6 Department Obstetrics and Gynecology, Regional Hospital H Hart, Tienen, Belgium; 7 Femicare vzw, Clinical Research for Women, Tienen, Belgium; Fred Hutchinson Cancer Center, United States of America

## Abstract

The application of high-throughput sequencing methods has raised doubt in the concept of the uniform healthy vaginal microbiota consisting predominantly of lactobacilli by revealing the existence of more variable bacterial community composition. As this needs to be analyzed more extensively and there is little straightforward data regarding the vaginal mycobiome of asymptomatic women we aimed to define bacterial and fungal communities in vaginal samples from 494 asymptomatic, reproductive-age Estonian women. The composition of the vaginal microbiota was determined by amplifying bacterial 16S rRNA and fungal internal transcribed spacer-1 (ITS-1) regions and subsequently sequencing them using 454 Life Sciences pyrosequencing. We delineated five major bacterial community groups with distinctive diversity and species composition. Lactobacilli were among the most abundant bacteria in all groups, but also members of genus *Gardnerella* had high relative abundance in some of the groups. Microbial diversity increased with higher vaginal pH values, and was also higher when a malodorous discharge was present, indicating that some of the women who consider themselves healthy may potentially have asymptomatic bacterial vaginosis (BV). Our study is the first of its kind to analyze the mycobiome that colonizes the healthy vaginal environment using barcoded pyrosequencing technology. We observed 196 fungal operational taxonomic units (OTUs), including 16 OTUs of *Candida spp.*, which is more diverse than previously recognized. However, assessing true fungal diversity was complicated because of the problems regarding the possible air-borne contamination and bioinformatics used for identification of fungal taxons as significant proportion of fungal sequences were assigned to unspecified OTUs.

## Introduction

Similar to most cavities and surfaces on the human body, the health and functioning of the vagina are closely linked to its microbial inhabitants. These microbes maintain adequate pH, help to prevent the acquisition of pathogens, stimulate the local innate immune system, and decrease symptoms and complications during pregnancies [1]–[3]. According to the ecological theory applied to all natural habitats, including microbial econiches within the human body, communities diverge from each other by two critical characteristics: taxon composition and physiological potential [4]. When these characteristics are altered, the functioning of the vaginal environment becomes disturbed and disease may emerge. The biggest enigma in recent years is determining how to define a normal state: how much variation within the prevailing vaginal microbial communities can still be considered within the normal boundaries. Originally, healthy vaginal microbiota was considered to be relatively non-diverse and consisted mainly of the *Lactobacillus* species, which produce lactic acid to maintain low vaginal pH and bacteriocins to kill potential pathogenic invaders [5]. Recent studies using cultivation-independent molecular methods, however, have shown that the composition of healthy vaginal microbiota may vary to a greater extent than was initially thought [6]–[8]. In particular, a significant proportion of asymptomatic, reproductive-age women are colonized by glucose-consuming species other than lactobacilli, such as *Megasphaera sp.*, *Atopobium vaginae*, *Gardnerella vaginalis*, *Prevotella sp.*, or *Streptococcus sp.*, even though these species have been linked to abnormal vaginal microbiota that are present in certain pathologic conditions, such as BV. Moreover, because BV affects up to 30% of all reproductive-age women in Western countries [9], any unusual shift in microbial communities should be addressed through further analysis. This also applies to some of the opportunistic fungal pathogens, such as *Candida albicans*, which can colonize approximately 20% of asymptomatic women [10]. Furthermore, even a slight modification in host defense and the composition of resident bacterial community can encourage the emergence of opportunistic infections caused by *C. albicans* [11]. Still, there is surprisingly little straightforward data regarding the presence of the *Candida* species and other fungi in the vaginal environment of asymptomatic women as well as the interactions and possible coexistence patterns with bacterial communities. The application of advanced molecular technologies and computational biology promises to increase our understanding regarding both the normal vaginal ecosystem as well as the different microbial diseases, especially those considered to be polymicrobial in origin [12].

In this study, using parallel pyrosequencing technology followed by taxonomic identification, we were able to define in detail the bacterial and fungal components of microbial communities present in the vaginal samples of asymptomatic reproductive-age women. In addition, we estimated the variation of normal vaginal microbial communities and linked those variations to medical history or lifestyle habits of the women.

## Materials and Methods

### Participants and sample collection

For this study, 494 healthy pre-menopausal Caucasian women, age 15–44 y (mean[±SD] 31.1 [±6.4] y), having a routine gynecological check-up visit at the Fertilitas private hospital, Estonia, from June to October 2010 were enrolled. Informed written consent was obtained from all participants prior to enrollment. In case of under-aged participants, verbal consent was obtained from one of their guardians by the doctor carrying out the sample collection and recruitment. We confined to verbal consent of the guardians because we expected the under-aged group of people to be old and aware enough to be sufficiently competent to make a decision about this study and thus no extra measures to document the approval of the guardians was taken. The study design (including the participation of under-aged women based on aforementioned terms) was approved by the Ethics Committee of Medical Research in Tallinn, Estonia. Exclusion criteria included pregnancy, menstruation, or any signs or symptoms of urogenital disorders. The presence of urogenital infections was excluded by inspection and *in speculo* examination before a sample was taken from the vaginal fornix and cervix using Rovers® Cervix-Brush® Combi (Rovers Medical Devices B.V. Oss, The Netherlands). Amsel criteria or Gram staining were not specifically applied for the exclusion of BV. The vaginal pH was measured during sampling with pH indicator strips (range 2.0–9.0; 0.5 unit increments; Merck & Co, Inc., USA). Collected samples were then stored at −20°C until batch analysis was performed.

Participants were asked to complete a questionnaire on their medical history and lifestyle habits, which specifically addressed their sexual behavior and gynecologic hygiene. The opinion of a gynecologist regarding physical appearance of the vaginal environment (e.g. redness, characterization of vaginal discharge etc.) was included with the questionnaire.

### DNA extraction

DNA was extracted from samples using a modified BioSprint 96 DNA Blood Kit (Qiagen, USA) protocol and Kingfisher Flex Magnetic Particle Processor (Thermo Scientific, Bremen, Germany). The BioSprint 96 DNA Blood Kit protocol was used with a pre-initial homogenization step using ceramic beads (Ø 0.4–0.6 mm, 0.5 g per sample; Saint-Gobain ZIRPRO, Le Pontet Cedex, France) and TissueLyser II (6 min at 30 Hz; Qiagen). In addition to samples collected from patients, negative controls were included to every separate extraction batch of 95 samples. Extracted DNA samples were stored at −20°C for further analysis.

### PCR amplification

Before pyrosequencing, the amplification of the desired sequence was carried out with two sequential PCR reactions. In the first PCR reaction, the V1-V2 hypervariable region of 16S rRNA genes (16S rDNA) was amplified with 8F and 357R broad range primers to assess the bacterial component of the vaginal microbiota [13]. The complete sequences of the primers were as follows: 8F-5′ TTGGCAGTCTCAGNNNNNNNN**AGTTTGATCCTGGCTCAG** 3′ and 357R-5′ GTCTCCGACTCAGNNNNNNNN**CTGCTGCCTYCCGTA** 3′. For assessment of the fungal component of the vaginal microbiota, the Internal transcribed spacer-1 (ITS-1) region of eukaryotic ribosomal DNA was amplified using ITS1F and ITS2 primers [14]. The complete sequence for the forward primer (ITS1F) was 5′ GTCTCCGACTCAGNNNNNNNN**CTTGGTCATTTAGAGGAAGTAA** 3′ and reverse primer (ITS2) was 5′ TTGGCAGTCTCAGNNNNNNNN**GCTGCGTTCTTCATCGATGC** 3′. Underlined letters indicate partial 454-specific sequencing adapters, bold letters denote amplicon-specific primer sequences (8F/ITS2 or 357R/ITS1F), and the 8 bp barcode is marked as 8 Ns, which refers to a unique sequence tag to barcode each sample [15]. Cycling parameters were 15 min at 95°C, followed by 5 cycles of 30 s at 95°C, 30 s at 50°C, and 60 s at 72°C, and then 35 cycles of 30 s at 95°C, 30 s at 65°C, and 60 s at 72°C, with a final extension at 72°C for 10 min.

To extend the partial 454-specific adapter sequences at both ends of each amplicon to full-length sequences, a second PCR was performed with full sequencing adapters (Primers A 5′-CCATCTCATCCCTGCGTGTCTCCGACTCAG-3′ and B 5′-CCTATCCCCTGTGTGCCTTGGCAGTCTCAG-3′) using the amplicon derived from the previous PCR step of the 16S rRNA gene or ITS-1 region amplification diluted 50-fold. Cycling parameters for the second PCR were 15 min at 95°C, followed by 5 cycles of 30 s at 95°C, 30 s at 62°C, and 60 s at 72°C, and then 20 cycles of 30 s at 95°C and 60 s at 72°C, with a final extension at 72°C for 10 min.

All PCR reactions were carried out in total reaction volume of 10 µL consisting of 5 µL Maxima Hot Start PCR Master Mix (Fermentas, Germany), 1 µL DNA template or water in case of negative controls added to every PCR reaction, and 0.2 µM each primer, with water added to reach the final volume. Reactions were performed on a Thermal cycler 2720 (Applied Biosystems, California, US).

### 454 pyrosequencing

Barcoded amplicons with full adapter sequences were pooled together by extraction of amplicons from a 1.5% agarose gel using the QIAquick Gel Extraction kit (Qiagen) and sequenced with the GS Junior Sequencing System (454 Life Sciences, Roche, Germany) according to the manufacturer's protocol.

### Analysis of bacterial sequences

Pyrosequencing noise was removed from the initial pre-trimmed dataset of the 16S rRNA gene V1-V2 region (based on “.qual” file output of 454 sequencing system), and only sequences longer than 170 bp were included for further processing. Reference sequences of aligned 16S rDNA were obtained from the SILVA ribosomal RNA database [16]. Operational taxonomic units (OTUs) with a 97% identity threshold were defined by the average neighbor hierarchical clustering algorithm using mothur 1.24.1 software [17]. To filter chimeric sequences, UChime was used by applying the “chimera.uchime” procedure from the mothur software in *de novo* mode, which first splits sequences into groups and then checks each sequence within a group using the more abundant groups as reference. For additional denoising, OTUs that had less than 5 sequences were removed. Taxonomic assignments were performed against the SILVA bacterial database using Ñaive Bayesian classifier with a confidence cutoff of 75% [18]. To represent sequences of the 10 most relatively abundant OTUs, an additional taxonomic assignment was carried out using BLASTN against the NCBI nt database (updated in May, 2012). Different OTUs assigned to the same species were numbered starting from the most relatively abundant OTU. For clustering of similar samples, we used a similarity tree based on hierarchical Yue-Clayton theta value [19] with a group cutoff of 0.30. Only clusters ≥15 members were included.

### Analysis of fungal sequences

Pyrosequencing noise was removed from the initial pre-trimmed dataset of ITS1 region (based on “.qual” file output of 454 sequencing system) and only sequences longer than 170 bp were included for further processing. The fungal sequences were first clustered with cd-hit-est software [20] with 97% cluster identity. Cd-hit-est chooses one representative sequence for each OTU (cluster), which can then be used for further analysis. In this current study, the cd-hit-est results were compared against the UNITE fungal database [21] using a BLAST search with 97% identity and an e-value below 10^−20^. In case of more than one match, the one that had the lowest e-value was kept for further analysis. OTUs that did not match the UNITE database were discarded. Similarly to the bacterial analysis, for additional denoising, only OTUs with more than 5 sequences were kept for further analysis. In addition, samples with less than 75 sequences were excluded from the dataset. The taxonomy for fungal OTUs was taken from the UNITE database.

### Statistical analysis

Statistics was performed using R 2.13.1 software and its packages: gplots [22], ggplot2 [23] and vegan [24]. The level of a significant difference was set at 5%. To describe general bacterial diversity, the Shannon diversity index was calculated. The Shannon index is a mathematical measure of species diversity in a community that accounts for both abundance and evenness of the species present. Taxonomic richness was also expressed through the number of observed OTUs. For reviewing the proportions of different bacteria, the relative abundances of OTUs identified within the same genus were summed and referred to as a summarized genus.

To analyze the correlation of medical history or lifestyle habits of the participants with Shannon diversity index values or summarized abundance of *Candida spp.*, a linear regression analysis was used. Before applying linear regression models, a correlation matrix was generated for all of the factors that were included into the models. If the Pearson's correlation coefficient was higher than 0.7 between the covariates, then one of them was removed. Both linear regression models included factors of gynecological diseases experienced recurrently and within the last 6 months prior to the sampling [BV, vulvovaginal candidosis (VVC), myco- and ureaplasmosis and no gynecological disease within the last 6 months], contraceptives (not used, condom, vaginal ring, contraceptive patch, hormonal pills, copper-containing intrauterine device, or levonorgestrel-releasing device), age of the participants, time from the last intercourse, characteristics of vaginal discharge (physiological, mucopurulent, homogeneous, white color or malodorous), and redness of the vaginal canal. In addition, models testing the correlation with the Shannon diversity index included factors of sexually transmitted diseases (STDs) experienced within the last 6 months prior to the sampling (gonorrhea, chlamydia, genital herpes, or no STDs) and characteristics of cervix uteri (conical or cylindrical shape, deformed, ovula Nabothi or with redness). The model testing the correlation with the summarized abundance of *Candida spp.* included only vaginal pH as an additional factor.

Linear regression analysis was also used for analyzing the correlation between the clustering of vaginal bacterial communities and vaginal pH, Shannon diversity index, and the number of observed bacterial OTUs. The prevalence of *C. albicans* among analyzed samples in relation to the relative abundance of the genera *Lactobacillus* and *Gardnerella* (comprised of the summarized relative abundance of OTUs belonging to aforementioned genera) was tested by a logistic regression model. All regression analyses were carried out with Holm-Bonferroni correction.

## Results

### Medical history and lifestyle habits of the participants

Among the 494 recruited asymptomatic Estonian women, the vaginal pH value varied in the range of 3–9, it was greater than 7 in 11 women. The mean value of vaginal pH was 4.7 (±0.8). Of the women assessed, 28.5% (n = 141) had experienced VVC and 10.5% (n = 52) had experienced BV repeatedly during their adult lives. During the last 6 months prior to the sampling, VVC was detected in 13.6% (n = 67) of the patients and BV in 5.7% (n = 28) patients respectively. Data regarding aerobic vaginitis are lacking, as this was not recognized as a separate entity by our clinicians in the current study.

Because there was no restriction on intercourse prior to the sampling, the last intercourse event was recorded as follows: 60 individuals (12.2%) had the last intercourse within 24 h prior to the sampling, 108 (21.9%) had sexual intercourse 24–48 h before sampling, 148 (30%) during the previous 7 d and 167 (33.8%) more than a week prior to the sampling. Twelve participants did not provide this information. A majority of participants (92.5%) reported themselves as heterosexual, while 13 (2.6%) reported themselves as homosexual and 25 participants (5.1%) did not answer this question.

### Vaginal bacterial communities

**Figure 1:**
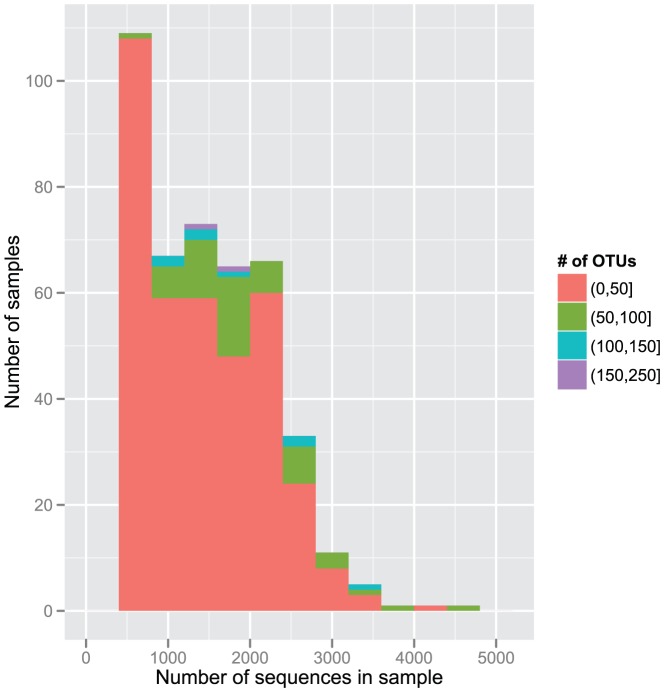
Distribution of 432 samples according to number of sequences and number of OTUs. The cutoff value is set to 400 sequences. Round and square brackets in the figure indicate to the exclusion and inclusion of adjacent value, respectively.

In total, we obtained 828,551 16S rRNA gene sequences with an average sequence read length of 300 bp. The number of sequences was higher than 400 in 432 samples, which were included for further analysis. Four hundred sequences per sample was the level whereby the rarefaction curves for 95% of the samples reached a 5% plateau. This means that increasing the number of sequences per sample did not increase the number of OTUs obtained in 95% of the samples (Fig. 1). After exclusion of these samples, the mean number of sequences per sample was 1330 (±818).

For further analysis, we only included OTUs (n = 208; FASTA file S1) that were present in at least one sample with a relative abundance of ≥1%. The additional cut-off was applied to normalize the filtering across samples with different sequences assigned to them and also to filter out artifacts generated by the limitations of laboratory workflow. The mean taxonomic richness (number of OTUs) per sample was 26.9 (±24). A comparison of these OTUs against the SILVA and NCBI databases showed that three of the OTUs were unclassified bacteria with an average summarized relative abundance of 0.01%. Lactobacilli accounted for the greatest proportion of the recovered OTUs (69.4%), followed by OTUs belonging to genera of summarized *Gardnerella* (11.2%), *Prevotella* (3.8%), *Atopobium* (2.2%), *Streptococcus* (1.5%), *Ureaplasma* (0.9%), *Escherichia coli* (0.5%), *Mycoplasma* (0.2%) and *Staphylococcus* (0.1%).

**Figure 2:**
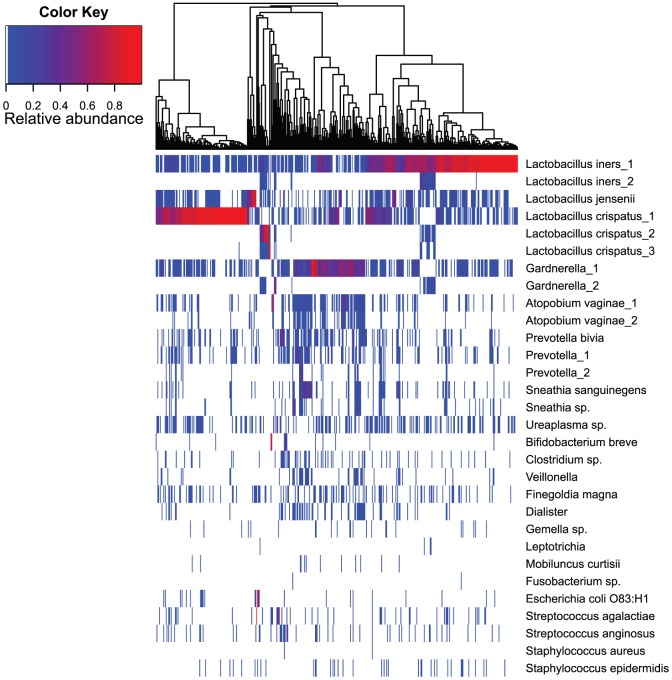
Relative abundance of bacterial OTUs found in the vaginal communities of healthy Estonian women (n = 432). Bacterial taxonomic assignments are indicated on the right of the heatmap at the Genus and Species level. The relative abundance is color coded and indicated by the color key on the left top of the map. The tree on the top of the heatmap characterizes the similarity of analyzed samples based on OTU composition of vaginal microbiota.

Lactobacilli were recovered from 98.8% of the samples, members of *Gardnerella* from 70.6%, *Prevotella* from 55.8%, *Ureaplasma* from 41%, *Atopobium* from 38%, *Streptococcus* from 30.6%, *Staphylococcus* from 13.9%, *Escherichia coli* from 6.5% and *Mycoplasma* from 4.6% of the samples. The relative abundance and prevalence of the most abundant and meaningful OTUs, which predominantly belonged to the aforementioned genera, is shown in Figure 2. Special emphasis should be laid on the OTU-s with identical names but different numbers. By visually analyzing the alignment of representative sequences of these OTU-s and ignoring the potential pyrosequencing errors, considerable similarities can be noted in all, except for *Prevotella* 1 and 2 (See Fig. S1).

### Bacterial community parameters in relation to the medical history and lifestyle habits

There was no correlation between the number of bacterial sequences of 16S rRNA gene retrieved per sample from the trimmed dataset and the Shannon diversity index of bacterial communities [correlation coefficient (CC) = −0.05], nor was there a correlation between the number of sequences per sample and the number of observed OTUs (CC = 0.36). These results confirmed that taxonomic richness was not created by the number of sequences by itself.

Linear regression analysis showed that the Shannon diversity index of vaginal bacterial communities increased with an increase in vaginal pH (P = 0.003) and with the presence of malodorous vaginal discharge (P<0.0001) and also that the Shannon diversity index was not influenced by other factors included to the analysis (P>0.5).

### Clustered bacterial communities

**Figure 3:**
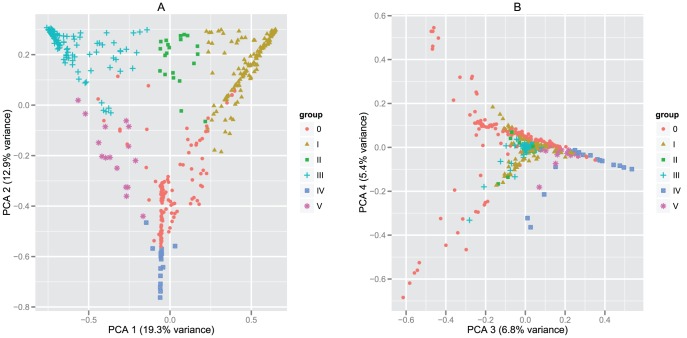
Two-dimensional (2D) plots describing the principal component analysis (PCA) of OTU composition among analyzed samples. The plots represent 2D projections of a multidimensional analysis where the relative abundance of each specific OTU defines a dimension. Both plots are the projection of the same analysis viewed at a different angle. The plots visualize the clustering and variability of studied vaginal bacterial communities. First (a) 2D plot of the first two PCA components describes the clustering of groups I–V. Second (b) 2D plot of third and fourth PCA component confirms that the samples belonging to non-classifiable group (0) are not clustering into separate or any other entity. The variance described by the respective PCA components (Axis-1 and Axis-2) is written in brackets.

**Figure 4:**
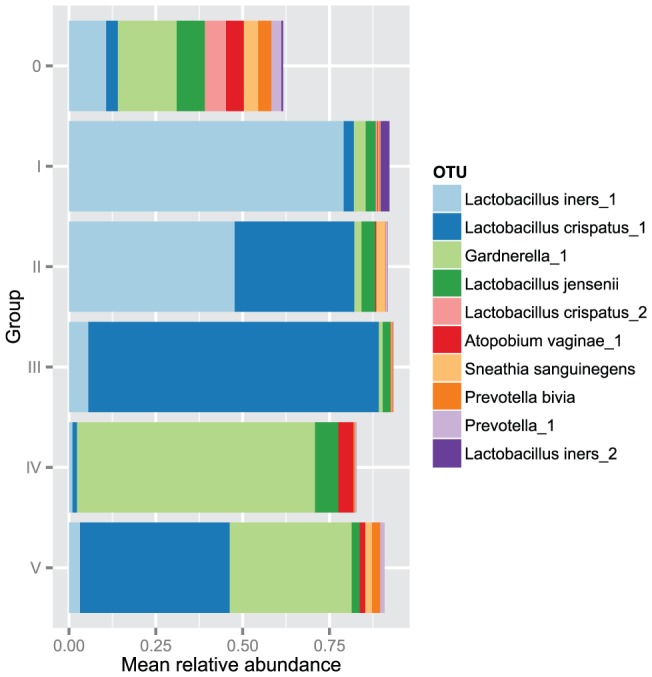
The distribution of 10 most relatively abundant OTUs in determined vaginal bacterial community groups. The distribution is presented based on mean relative abundance values (x-axis) of these OTU-s among bacterial community groups (y-axis).

Three hundred and seven (71.1%) vaginal bacterial communities clustered into 5 major groups based on the similarities in relative abundance values of bacterial OTUs (OTU composition) (Fig. 3). Each group harbored ≥15 members. The remaining vaginal communities (N = 125; 28.9%) could not be classified in any of the 5 groups and were rather erratic, non-related, heterogeneous communities that did not cluster into any other entity (hereafter referred to as the non-classifiable or 0-group). OTUs belonging to members of lactobacilli dominated four out of five groups. In two of the groups (I and II), the most abundant OTU was *Lactobacillus iners*_1, and in two other groups (III and V) *L. crispatus_1* was the most abundant. In group IV, lactobacilli did not have the most abundant OTUs, while in this group, the predominant bacterium was *Gardnerella*_1 (Table 1; Fig. 4).

**Table 1:** Mean (±SD) values of selected parameters of vaginal environment within bacterial community groups.

*Community groups*	*Group size*	*Vaginal pH*	*Shannon diversity index*	*Number of observed OTUs*	*Dominating OTUs in bacterial community (relative abundance/prevalence)*
**I**	**145**	4.58(±0.9)***	0.8(±0.6)***	26.1(±27.4)***	*Lactobacillus iners_*1 (79.1%/100%)
					*Gardnerella_ 1* (3.4%/52.4%)
					*Lactobacillus crispatus_1* (3.0%/59.3%)
**II**	**21**	4.54(±0.6)*	1.4(±0.5)**	34.2(±24.7)	*Lactobacillus iners_1* (47.7%/100%)
					*Lactobacillus crispatus_1* (34.4%/100%)
					*Lactobacillus jensenii* (4.0%/81.0%)
**III**	**108**	4.19(±0.5)***	0.7(±0.4)***	20.4(±14.8)***	*Lactobacillus crispatus_1* (83.7%/100%)
					*Lactobacillus iners_1* (5.6%/80.6%)
**IV**	**17**	4.88(±0.6)	1.1(±0.4)***	24.9(±11.5)*	*Gardnerella_1* (68.5%/100%)
					*Lactobacillus jensenii* (6.7%/35.3%)
					*Lactobacillus gasseri* (5.7%/47.1%)
**V**	**16**	4.64(±0.7)	1.4(±0.4)*	20.6(±11.1)**	*Lactobacillus crispatus_1* (43.1%/100%)
					*Gardnerella_1* (35.1%/100%)
					*Lactobacillus iners_1* (3.2%/56.3%)
**0**	**125**	5.02(±0.9)	1.8(±0.6)	39.4(±27.8)	

Dominating OTUs are listed as top 3 with the mean relative abundance at least 3%. Linear regression models were used to test the differences between the groups (I–V) and 0-group:

*** P<0.001;

** P = 0.01–0.001;

* P = 0.01–0.05.

Among the non-classifiable group, there were no dominating bacteria that had relative abundance over 20%, but the prevalence of the following OTUs was relatively high: *Gardnerella_1* (74.1%) and members belonging to genera *Prevotella* (69.2%), *Atopobium vaginae*_1 (52.4%), *Ureaplasma sp*. (33.6%), *Sneathia sanguinegens* (30.1%) and *Clostridium sp.* (16.8%). A comparison of the community groups (I–V) with 0-group in linear regression analysis revealed significant differences in the mean bacterial diversity expressed by the Shannon diversity index, in the mean number of observed OTUs, and in the mean values of vaginal pH (Table 1).

### Vaginal fungal communities

From 494 collected samples, we analyzed fungal communities in 251 samples. In total, we obtained 276,336 ITS-1 region specific sequences and the average sequence read length was 220 bp. The count of sequences was higher than 75 in 216 samples, which were included for further analysis. Seventy-five sequences per sample was the level whereby the rarefaction curves for 95% of the samples reached a 5% plateau. After this exclusion of the samples, the mean number of sequences per sample was 1076.7 (±1282.2). Only OTUs (n = 196; FASTA file S2) that were present in at least one sample with a relative abundance ≥1% were included for further analysis. The additional cut-off was applied to normalize the filtering across samples with different sequences assigned to them and also to filter out artifacts generated by the limitations of laboratory workflow. The mean taxonomic richness per sample was 7.8 (±4). A comparison of these OTUs against the UNITE database resulted in 161 unique hits at the species taxonomic level.

The mean relative abundance of *Candida spp.* was 36.9%. We were able to detect 16 different OTUs belonging to genus *Candida*: *C. parapsilosis* (n = 1), *C. dubliniensis* (n = 1), *Candida sp.* VI04616 (n = 1), *Pichia kudriavzevii* (*Candida krusei*) (n = 3) and *C. albicans* (n = 10). The relative abundance of summarized *C. albicans* was 34.1%, *P. kudriavzevii* 2.3%, *C. parapsilosis* 0.3%, *Candida sp.* VI04616 0.3% and *C. dubliniensis* 0.04%. OTUs belonging to genus *Candida* were detected in 69.9% of samples. The prevalence of *C. albicans* was 67.6%, *P. kudriavzevii* 9.7%, *C. dubliniensis* 0.5% and both *C. parapsilosis* and *Candida sp.* VI04616 1.9% of 216 samples analyzed. The prevalence and summarized relative abundance of all predominant fungal OTUs are shown in Table 2. A considerable number of these OTUs were unspecified (uncultured OTUs that did not have a taxonomic assignment lower than kingdom), and their summarized relative abundance was 38.6%. The prevalence of these OTUs was 85.2% of samples.

**Table 2:** The prevalence and summarized relative abundance of predominant fungal taxa.

*Division*	*Order*	*OTU*	*Prevalence*	*Summarized relative abundance*
*Ascomycota*			0.87	0.58
	*Saccharomycetales*		0.74	0.37
		*Candida albicans*	0.68	0.34
		uncultured *Saccharomycetales*	0.57	0.06
		*Pichia kudriavzevii (C. krusei)*	0.10	0.02
	*Capnodiales*		0.32	0.05
		*Davidiellaceae sp* PIMO_97	0.21	0.03
		*Cladosporium perangustum*	0.13	0.01
		*Cladosporium sp* BMP2897	0.02	0.01
		uncultured *Epicoccum*	0.06	0.01
	*Eurotiales*		0.21	0.03
		*Eurotium amstelodami*	0.06	0.01
	*Pleosporales*		0.16	0.03
		*Alternaria alternata*	0.06	0.01
	*Helotiales*		0.04	0.01
*Basidiomycota*			0.27	0.03
	*Sporidiobolales*		0.03	0.01
		*Rhodotorula sp* LH51	0.02	0.01

Only taxons with ≥1% relative abundance and occurring in more than 1% of the tested samples were included. Unspecified OTUs are excluded from the table.

### Fungal community parameters in relation to the medical history, lifestyle habits, and bacterial community parameters

**Figure 5:**
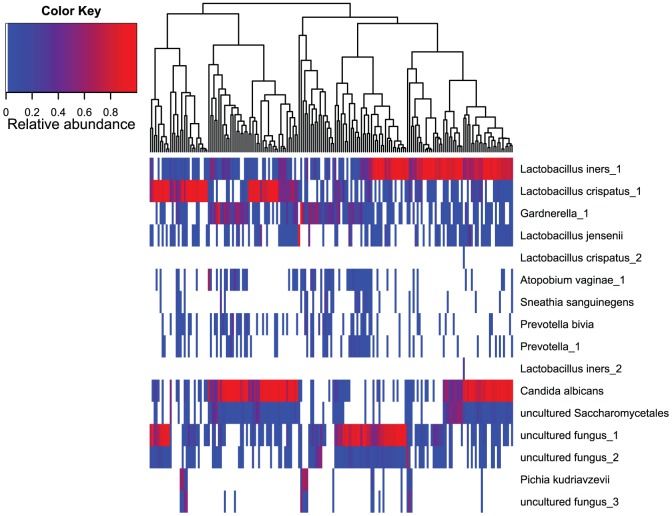
Relative abundance of the most abundant bacterial and fungal OTUs found in the vaginal communities of 181 women. Taxonomic assignments are indicated on the right of the heatmap at the Genus and Species level. The relative abundance is color coded and indicated by the color key on the left top of the map. The tree on the top of the heatmap characterizes the similarity of analyzed samples.

There was no correlation (CC = −0.05) between the number of sequences per sample and the calculated Shannon diversity index for fungal communities, indicating that taxonomic richness was not created by the number of sequences by itself. Linear regression analysis incorporating different factors of medical history and lifestyle habits did not reveal any correlation between these factors and summarized abundance of *Candida spp.* (P>0.05). Also, the relative abundance values of lactobacilli and *Gardnerella* were not correlated with the prevalence of *C. albicans* (P>0.05) when tested by logistic regression analysis. Figure 5 shows the relative abundance of the most abundant microbes (10 bacteria and 6 fungi) among 181 samples that had both the 16S rRNA gene and ITS-1 sequencing data available. When analyzing the heatmap patterns, three pairs of fungal OTU-s were almost identically distributed between the 181 samples. These OTU pairs were *C. albicans* and uncultured *Saccharomycetales*; uncultured fungus_1 and uncultured fungus_2; and *Pichia kudriavzevii* and uncultured fungus_3. By visually analyzing the alignment of representative sequences of these OTU-s, there were considerable similarities between uncultured fungus_1 and uncultured fungus_2; and *Pichia kudriavzevii* and uncultured fungus_3 but not between *C. albicans* and uncultured *Saccharomycetales* (Fig. S2).

## Discussion

This study is the first to analyze vaginal microbial communities of Estonian women by using parallel pyrosequencing technology. We have shown that the composition of vaginal microbiota varies widely among healthy women with homogeneous ethnic origin. According to their taxonomic composition, the majority of bacterial communities clustered into five major community groups that were characterized by significantly different bacterial diversity, whereas a considerable number of communities (28.9%) did not form differentiated groups. As the women participating in the study were asymptomatic, it is not surprising that four major vaginal microbial community groups were dominated by the *Lactobacillus* species (*Lactobacillus iners* and *L. crispatus*). In general, 99% of the women were colonized with lactobacilli, and the mean relative abundance of this genus was 70%. This is consistent with the results of Srinivasan et al., who also found that women without BV have vaginal microbiotas dominated by *Lactobacillus* species, notably either *L. crispatus* or *L. iners* [25]. Nevertheless, members of genera suggestive of a “BV-like” microbiota, such as *Gardnerella* and *Atopobium*, were also relatively abundant in clustered community groups of our results.

Detection of *Gardnerella vaginalis* and *Atopobium vaginae* in vaginal samples has been historically related to vaginal microbiota that is shifted towards a diseased state [26]–[28]. The study by Srinivasan et al. associated these species with several clinical criteria used in the diagnosis of BV and they also described relatively strong co-occurrence of these species [25]. However, previous studies have also shown that *G. vaginalis* could be detected in approximately 50% of vaginal microbial communities belonging to healthy women [29], [30]. In our study, 70.6% of the women were colonized with OTUs belonging to genus *Gardnerella*, which supports these recent results. Similar to the genus *Gardnerella*, OTUs belonging to genera *Prevotella* and *Atopobium* were widespread among healthy women participating in our study (prevalence 55.8% and 38% of samples, respectively), which is in concordance with recent studies showing *A. vaginae* and *Prevotella spp.* to be prevalent in the healthy vaginal environment [6], [7].

Additionally, aerobic bacteria, such as members of the genera *Streptococcus* and *Staphylococcus*, as well as *Escherichia coli*, were detected in healthy vaginal microbiota. The abundance of these microbes was low (<2%), but the prevalence among participating women was relatively high, reaching 30.6% for streptococci. All of these bacteria have been linked to aerobic vaginitis [31].

Also, of note, *Ureaplasma sp.* was present in 41% of the studied vaginal microbial communities, but with very low abundance (0.9%), indicating its affiliation with normal vaginal microbiota. However, this does not rule out the possibility that *Ureaplasma sp.* can cause disease when present in high numbers.

Broad taxonomic variation and clustering of vaginal communities has been performed before in large scale studies that have analyzed the composition of normal vaginal microbiota in ethnically diverse participants, including Asian, Hispanic, African American, and Caucasian women from the United States and Japanese women from Japan. In those studies, most of the bacterial community groups were dominated by *Lactobacillus* species (*L. crispatus*, *L. gasseri*, *L. iners*, and *L. jensenii*), but the studies also found that a number of communities were dominated by other genera, such as *Prevotella*, *Atopobium*, and *Gardnerella* [6], [8], [32]. Interestingly, the number of vaginal microbial communities with miscellaneous community profiles accounted for as much as 29% of our study group, which is very similar to the results of Ravel *et al.*, who found heterogeneous community profiles in 27% of healthy women studied in the United States [6].

Different community groups may represent different stages in the menstrual cycle, as the shifts in vaginal microbiota in relation to the menstrual cycle have been previously reported [33], [34]. Srinivasan *et al.*, who used qPCR to evaluate levels of key vaginal bacteria, demonstrated elevated levels of *G. vaginalis* and *L. iners* in the vaginal environment during menstruation, whereas levels of *L. jensenii* and *L. crispatus* decreased during that time [33]. The shifts in the composition of vaginal microbiota during the menstrual cycle were observed by Gajer *et al.*, who showed that the fluctuations in the community composition were mainly affected by time in the menstrual cycle and community class (classification was based on clustering done by Ravel *et al.* [6]) [34]. However, the fluctuation of community composition related to the menstrual cycle did not occur in all cases.

Among asymptomatic women participating in our study, microbial diversity increased with higher vaginal pH values and was also higher when a malodorous discharge was present. High pH values and malodorous discharge have both been related to BV before [35]. Moreover, studies such as by Srinivasan et al. have shown that patients suffering from BV have higher bacterial diversity and richness than individuals without BV [25]. Asymptomatic BV is relatively common, as approximately half of the women who are diagnosed with BV are asymptomatic [35] and could have been excluded from the cohort of current study by applying Amsel diagnostic criteria and Nugent scoring. As this was not done and the exclusion factors included only symptomatic urogenital disorders, women having asymptomatic BV were probably not excluded from our study. The abundant presence of asymptomatic BV and other abnormal vaginal microbiota types has been recognized for decades and is a continuing concern to clinicians, as these microbiota types are associated with diverse adverse outcomes during pregnancy (such as preterm delivery and chorioamnionitis [3], [36]) and increased risk of HIV and other STD transmission [2], [37]. Moreover, the simple treatment of BV with metronidazole does not prevent these complications [3]. On the other hand, because these women with more diverse vaginal microbiota in combination with higher vaginal pH or malodorous discharge did not seem to have a reduced quality of life, one can question the current diagnostic strategies and the application of therapy in the absence of symptoms.

Although yeast infections are frequent and can disturb the vaginal microbiota dramatically, studies addressing vaginal microbiota have mainly focused on bacterial inhabitants, while discarding information about the vaginal mycobiome. Furthermore, studies analyzing vaginal fungal colonization have mostly focused on the *Candida* species only in the context of vaginal pathology [38]. The variation of fungal taxa in the vaginal environment may not be limited to a few known yeasts, such as *Candida* and *Saccharomyces* species. Ghannoum *et al.* used barcoded pyrosequencing techniques to analyze the oral mycobiome and found a remarkably higher prevalence of fungal taxons during mouth rinsing than shown before with culture-based methods [39]. Our study is the first of its kind to analyze the mycobiome that colonizes the healthy vaginal environment using barcoded pyrosequencing technology. Our results showed vaginal colonization with *Candida spp.* in 64.5% of patients, which is considerably higher than the prevalence of 20% found in asymptomatic healthy women in earlier studies [40]. 82% of the fungal OTUs identified as *Candida spp.* belonged to *C. albicans*, which is also in full accordance with earlier studies.

*C. parapsilosis*, *C. dubliniensis*, *Candida sp.* VI04616, and *Pichia kudriavzevii* (*Candida krusei*) were the only non-*albicans Candida* OTUs detected in our study. All aforementioned species, with the exception of *Candida sp.* VI04616, have been linked to vaginal pathology in previous studies. *C. parapsilosis* can cause VVC and leads to symptomatic disease in two thirds of colonized women [41]. *C. dubliniensis* is mostly an opportunistic pathogen in the oral cavity of immunocompromised individuals, but it has recently been isolated from vaginal samples collected from women suffering from VVC in Turkey [42]. *Pichia kudriavzevii* (*Candida krusei*) has been reported to cause chronic vaginitis in older women, which is notably resistant to fluconazole [43]. Nevertheless, women participating in our study were all asymptomatic, indicating that *C. parapsilosis*, *C. dubliniensis*, and *P. kudriavzevii* can be affiliated with normal vaginal microbiota without causing disease.

When analyzing the fungal non-*Candida* OTUs we came across several complications. First, most of the abundant fungal non-*Candida* OTUs were identified as potential air-borne contaminants (e.g. OTUs belonging to genus *Cladosporium*) as seen in Table 2. This may be an indication of contamination problem facing the studies analyzing fungal diversity with highly sensitive methodology, although the workflow in current study was carried out under relatively stringent conditions (DNA was extracted under laminar flow cabinet and PCR reactions were prepared under UV PCR cabinets) and the negative controls included to the process in DNA extraction and PCR reaction stage did not give a positive signal with ITS-1 specific PCR. Thus, stricter conditions for sample collection and/or lowering the number of PCR cycles used may solve or reduce this issue. Second, the summarized relative abundance of unspecified OTUs was 38.6%. The failure to specify the strains is due to two problems; the information about vaginal fungi is under-represented in sequence databases used in our study (the NCBI-based UNITE database). Also, the clustering method of cd-hit appears to be relatively imprecise for metagenomic identification of fungal OTUs. As shown in Figure 5, several fungal OTUs shared almost identical heatmap patterns, suggesting an almost identical prevalence of these OTUs among the studied samples. The probability for this kind of distribution to happen by chance or have a biological meaning is low, and therefore these OTUs are most likely bioinformatic or sequencing artifacts of a single OTU (this is supported by the alignment analysis revealing substantial sequence similarities in majority of these kinds of OTU pairs). In that case, it suggests that there is an over-estimation of the actual fungal OTU number, but the over-estimation is not considerable, because the UNITE database search resulted in 161 unique hits. Importantly, this number is not considerably smaller than the overall number of detected fungal OTUs (n = 196). We defined fungal OTUs by widely used similarity level of 97%, but lowering this value to 96% or lower may offer a solution to this bias.

The limitation of primer efficiency can be another factor that may be responsible for biases in the results. This may be problematic when comparing results with other studies. The primer pair chosen by us to amplify V1-V2 region of 16S rRNA gene has been suggested to be suitable for community clustering and taxonomic assignments in a wide range of datasets based on bioinformatic analysis [44]. Nevertheless, it has been shown that the primer-binding sites of 27F (8F) primer have several sequence variations and mismatches against several bacteria, including *Gardnerella vaginalis* [45]. Also, ITS region is widely used for studies on fungal diversity, but most of the primers used to amplify different parts of ITS have several disadvantages that may bias the results of these diversity studies [46]. For instance, the primer pair used in current study is shown to be fungal specific but on the other hand has relatively low coverage of fungal sequences belonging to taxonomic groups of *Ascomycete* and *Basidiomycetes* under strict PCR conditions [46].

In conclusion, we have presented the first large-scale study addressing the vaginal microbial community composition of healthy Estonian women while concomitantly considering the mycobiome. We were able to confirm the findings of previous analogous studies assessing the composition of healthy vaginal microbiota by observing the clustering of microbial profiles into five distinctive community groups. Finally, the fungal component of these communities is more diverse than expected, although the bioinformatics for identification of these taxons is still currently incomplete.

## Supporting Information

Figure S1 Alignment of representative sequences of *Lactobacillus iners_1* and *L.iners_2* (A); *L.crispatus_1*, *L.crispatus_2* and *L.crispatus_3* (B); *Gardnerella_1* and *Gardnerella_2* (C); *Atopobium vaginae_1* and *A.vaginae_2* (D); and *Prevotella_1* and *Prevotella_2* (E), respectively. Alignment was carried out with Muscle 3.8.31 using default parameters except for the gap value, which was −4 in current study.
(EPS)

Figure S2 Alignment of representative sequences of *C. albicans* and uncultured *Saccharomycetales* (A); uncultured fungus_1 and uncultured fungus_2 (B); and *Pichia kudriavzevii* and uncultured fungus_3 (C), respectively. Alignment was carried out with Muscle 3.8.31 using default parameters except for the gap value, which was −4 in current study.
(EPS)

FASTA file S1 **List of all bacterial OTU-s and their representative sequences present in the dataset of current study.** Taxonomy assignments are based on SILVA database.
(FNA)

FASTA file S2 **List of all fungal OTU-s and their representative sequences present in the dataset of current study.** Taxonomy assignments are based on UNITE database.
(FNA)
